# Infant gut microbiota and the hygiene hypothesis of allergic disease: impact of household pets and siblings on microbiota composition and diversity

**DOI:** 10.1186/1710-1492-9-15

**Published:** 2013-04-22

**Authors:** Meghan B Azad, Theodore Konya, Heather Maughan, David S Guttman, Catherine J Field, Malcolm R Sears, Allan B Becker, James A Scott, Anita L Kozyrskyj

**Affiliations:** 1 Department of Pediatrics, University of Alberta, 3-527 Edmonton Clinic Health Academy 11405 – 87th Avenue, Edmonton, AB, T6G IC9, Canada; 2 Dalla Lana School of Public Health, University of Toronto, Toronto, ON, Canada; 3 Cell & Systems Biology, University of Toronto, Toronto, ON, Canada; 4 Agriculture, Food & Nutritional Sciences, University of Alberta, Edmonton, AB, Canada; 5 Department of Medicine, McMaster University, Hamilton, ON, Canada; 6 Pediatrics & Child Health, University of Manitoba, Winnipeg, MB, Canada; 7 Manitoba Institute of Child Health, Winnipeg, Canada

**Keywords:** Infants, Gut microbiota, Gut microbiome, Hygiene hypothesis, Microflora hypothesis, Pets, Siblings, Atopy, Allergic disease, Environmental exposures

## Abstract

**Background:**

Multiple studies have demonstrated that early-life exposure to pets or siblings affords protection against allergic disease; these associations are commonly attributed to the “hygiene hypothesis”. Recently, low diversity of the infant gut microbiota has also been linked to allergic disease. In this study, we characterize the infant gut microbiota in relation to pets and siblings.

**Methods:**

The study population comprised a small sub-sample of 24 healthy, full term infants from the Canadian Healthy Infant Longitudinal Development (CHILD) birth cohort. Mothers reported on household pets and siblings. Fecal samples were collected at 4 months of age, and microbiota composition was characterized by high-throughput signature gene sequencing.

**Results:**

Microbiota richness and diversity tended to be increased in infants living with pets, whereas these measures were decreased in infants with older siblings. Infants living with pets exhibited under-representation of Bifidobacteriaceae and over-representation of Peptostreptococcaceae; infants with older siblings exhibited under-representation of Peptostreptococcaceae.

**Conclusions:**

This study provides new evidence that exposure to pets and siblings may influence the early development of the gut microbiota, with potential implications for allergic disease. These two traditionally protective “hygiene hypothesis” factors appear to differentially impact gut microbiota composition and diversity, calling into question the clinical significance of these measures. Further research is required to confirm and expand these findings.

## Background

In recent decades, dozens of studies from around the world have reported that early-life exposure to pets or older siblings is protective against the development of allergic disease [1-4], and these associations are commonly attributed to the “hygiene hypothesis”. In its original form, this hypothesis claims that limiting early-life infection impedes natural immune system development and causes predisposition to allergic disease [5]. The modified “microflora hypothesis” proposes that, rather than specifically limiting infection, the overly hygienic Western lifestyle limits general microbial exposure and alters the colonization of the infant gut, which in turn disrupts development of the immune system and ultimately leads to allergic disease [6].

In support of the microflora hypothesis, reduced gut microbiota diversity during infancy has been associated with allergic disease later in childhood [7-13]. One could speculate that this is a function of fewer microbes in the home environment; however, human and piglet studies indicate that greater microbe diversity in the environment actually leads to reduced diversity of the gut microbiota [14,15]. In some cases, the prevalence or relative abundance of specific organisms has been associated with atopic outcomes. For example, early-life colonization by *Clostridium difficile* reportedly increases risk for childhood wheeze, eczema and asthma [16], whereas certain Bifidobacteria and Lactobacilli are considered protective [8,17-19]. It therefore remains unclear whether *diversity* is paramount, or whether gut microbiota *composition* is more important for immune system development and avoidance of atopic disease.

Household pets and siblings are thought to protect against atopy by increasing contact with environmental microbes during early life, thereby promoting a diverse and healthy gut microbiota. While associations of pets and siblings with atopy have been reported (and debated) for decades [1-5,20,21], few studies have directly evaluated the gut microbiota as a mediator. There is only limited evidence that contact with siblings influences the gut microbiota [22,23], and no studies have evaluated the role of pets.

In this study, we aimed to determine the impact of two early-life exposures traditionally associated with the hygiene hypothesis, pets and siblings, on the diversity and composition of infant gut microbiota.

## Methods

### Study design

This descriptive study of 24 infants represents a subset of the larger CHILD (Canadian Healthy Infant Longitudinal Development) national population-based birth cohort (http://www.canadianchildstudy.ca). Participants were enrolled in Winnipeg, Manitoba between November 2008 and August 2009. Mothers completed standardized questionnaires which addressed household pets, siblings, breastfeeding, infant medications, maternal atopy and education. Mode of delivery was obtained from hospital birth records. Written informed consent was obtained from parents at enrollment. This study was approved by the University of Manitoba Human Research Ethics Board.

### Sample collection, DNA extraction and amplification

Fecal samples were collected from 3–4 month old infants as part of a scheduled home visit. Samples were refrigerated during transport and then stored at −80°C. Whole genome DNA was extracted from 40 mg of stool using the FastPrep DNA for Soil Kit (MP Biomedicals Inc, Solon, OH, USA) [24]. The bacterial 16S rRNA gene, hypervariable regions V5-V7, was amplified through PCR using primers optimized for gut-occurring taxa: 5′-GGGKAKCRAACVGGATTAGATACCCBGGTAGTCCWNRCHSTAAACGDTG-3′(mV5 + 791) and 5′-GGSCRTRMKGAYTTGACGTCRYCCCCDCCTTCCTCC-3′ (V7-1104). Optimization was achieved by modifying previously-described primers [25] to accommodate a polymorphism unique to the gut-associated genus *Bifidobacterium*; the modified base is underlined in the primer sequence above. The primers were barcoded so that each sample could be uniquely identified post-sequencing. Each PCR mixture (50 μl) contained 5 μl 10X Hotstart Buffer, 400 μM dNTPs, 1.5 mM MgCl_2_, 2.5Uof Hotstart Taq polymerase (Fermentas, Glen Burnie, MD, USA), 0.02 mg Ultrapure Bovine Serum Albumin (Ambion, Austin TX), molecular biology reagent grade water (Sigma-Aldrich, St. Louis, MO, USA) , 0.16 μM primer, and 2 μl bacterial template DNA (10 ng/μl). The PCR program consisted of an initial DNA denaturation step at 94°C (4 min), followed by 18 cycles of DNA denaturation at 94°C (45 sec), an annealing step at 56°C (30 sec) and an elongation step at 72°C (2 min 30 sec), and was performed on the PTC-200 Peltier Thermal Cycler (MJ Research, St. Bruno, QC, Canada). The PCR product was cleaned with GENECLEAN® Turbo Kit (MP Biomedicals Inc, Solon, OH, USA) and gel purified using the E-gel® SizeSelect™ 2% agarose gel cutting system (Invitrogen, Carlsbad, CA, USA). Fifty nanograms of cleaned/extracted product from each sample was combined and concentrated for sequencing, using an Amicon® Ultra-4 30 K centrifugal filter (Millipore, Billerica, MA, USA).

### 16S rRNA sequencing and taxonomic classification

Pooled PCR amplicons were sequenced at portions of the V5, V6, and V7 16S rRNA hypervariable regions using Serial Illumina Sequencing (SI-Seq) [25] at the University of Toronto Centre for the Analysis of Genome Evolution & Function (CAGEF).

Reads were concatenated for a final length of 144 bp, and then processed through the SI-Seq analysis pipeline for de-barcoding and quality filtering, which removed reads having more than 10 sites with a Phred score less than 20. The resulting high quality reads were denoised, cleared of chimeras, and clustered into operational taxonomic units (OTUs) using the otupipe scripts [26]. An empirically derived nucleotide identity threshold of 87% was used for OTU clustering [25]. One representative sequence from each OTU was classified according to the SILVA taxonomy by 95% identity (i.e., genus level) clustering with the SILVA database sequences [27] formatted to SI-Seq read structure. Within each sample, OTUs with abundances lower than 0.18% were removed from the analysis based on an empirically derived misclassification/sequencing error rate [25]. After cleaning and processing, a total of 2.86 million reads were retained (median 9.6 × 10^4^ per sample, range 1.5 × 10^4^ – 4.5 × 10^5^).

### Quantitative PCR

We followed the method of Penders et al. for qPCR analysis of *Clostridium difficile*[23]. Oligonucleotides were manufactured by IDT (Integrated DNA Technologies Inc, Coralville, IA, USA). All reactions were performed on the MiniOpticon™ Real-Time PCR System (Bio-Rad, Hercules, CA, USA).

### Statistical analysis

In this descriptive paper, we report fecal microbiome biodiversity and relative abundance of bacterial taxa according to household pets and siblings. Biodiversity measures, the Chao1 estimator of species richness and the Shannon diversity index [28], were calculated using QIIME (http://qiime.org/) [29] with rarefied data (10,000 sequences per sample). These normally-distributed index measures were compared by two-sided t-test. Differential abundance of bacterial taxa was assessed at the family and genus levels using Metastats (http://metastats.cbcb.umd.edu). Distribution of potential confounding variables according to pets or siblings was assessed by chi-squared test or Fisher’s exact test, as indicated.

## Results

### Study population

Fecal samples were collected from 24 healthy full-term infants (mean age 17.4 ± 3.2 weeks). The study population (Table 1) comprised equal numbers of males and females. Thirteen infants (54%) had at least one older sibling and 15 (63%) lived in a household with pets (at least one cat or dog). At the time of sampling, 15 (63%) infants were exclusively or partially breast-fed. Six infants were delivered by caesarean section and 3 infants had received antibiotics. There was no significant difference in the rate of breastfeeding, antibiotic use or caesarean delivery according to the presence of household pets or siblings.

**Table 1:** Characteristics of study subjects

	**Overall (N = 24)**		**Households with pets (N = 15)**		**Households with siblings (N = 13)**	
N (%):						
Gender; male	12	(50.0)	6	(40.0)	7	(53.9)
Caesarean Delivery	6	(25.0)	3	(20.0)	3	(23.1)
Breastfed	15	(62.5)	8	(53.3)	9	(69.2)
Maternal Atopy	9	(37.5)	6	(40.0)	6	(46.2)
Siblings	13	(54.2)	6	(40.0)	-	-
Pets	15	(62.5)	-	-	6	(46.2)
Infant antibiotics	3	(12.5)	2	(15.4)	1	(8.3)
Maternal PS Education	18	(75.0)	13	(86.7)	9	(69.2)
Mean (SD):						
Birth Weight; g	3441	(493)	3453	(551)	3455	(357)
Gestational Age; wks	39.2	(1.6)	39.3	(1.4)	39.5	(1.2)

SD, standard deviation; PS, post-secondary. Comparisons by chi-squared test / Fisher's exact test, or t-test; no statistically significant differences observed.

### Fecal microbiota composition

**Figure 1:**
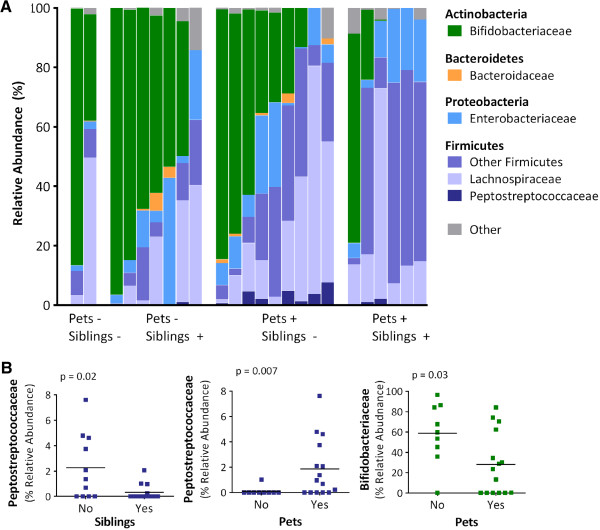
**Fecal microbiota composition for 24 infants (mean age 4 months) according to the presence of household pets and siblings. A**) Relative abundance of dominant bacterial families; each column represents one infant. **B**) Selected differentially abundant families according to pets and siblings; bars indicate means. Relative abundance determined by 16S rRNA sequencing; comparisons conducted with Metastats (see Methods and Table 2).

The relative abundance of dominant bacterial groups for each infant, as detected by 16S rRNA sequencing, is shown in Figure 1A. Infant fecal microbiota were generally dominated by Actinobacteria (median 36.4%, mainly the genus *Bifidobacterium*) and/or Firmicutes (median 43.8%, with diverse representation from numerous genera). Proteobacteria were less abundant (median 7.4%), though present in all subjects, whereas Bacteroidetes were detected in less than half (37.5%) of the study population.

Despite high inter-subject variability, we were able to detect significant effects of exposure to pets and siblings on the relative abundances of several bacterial taxa (Table 2 and Figure 1B). We found that infants living with pets had significant over-representation of Clostridiaceae, *Veillonella* (especially for dogs), Peptostreptococcaceae and *Coprococcus*. Bifidobacteria were under-represented among infants living with pets (especially for cats). For dogs only, there was also under-representation of *Eggerthella*. Fewer differences were detected according to siblings; however, Peptostreptococcaceae were significantly under-represented among infants with older siblings. Similar trends were observed when examining the prevalence (rather than relative abundance) of bacterial taxa (Additional file 1: Table S1). For example, Peptostreptococcaceae were detected significantly more often among infants with pets compared to those without (67% vs. 11%, p = 0.01). Stratified statistical comparisons of pets by siblings were not pursued due to small group sizes; however, we observed that infants exposed to both pets and siblings tended to have particularly low relative abundance of Bifidobacteriaceae (Figure 1A).

**Table 2:** Relative abundance of dominant bacterial taxa in infant stool, according to household pets and siblings

**Taxon**	**Overall**		**Older siblings**			**Pets**			**Dog**			**Cat**		
	**N = 24**		**No**	**Yes**	**p**	**No**	**Yes**	**p**	**No**	**Yes**	**p**	**No**	**Yes**	**p**
	Mean	(SD)	n = 11	n = 13		n = 9	n = 15		n = 12	n = 12		n = 17	n = 7	
**Actinobacteria**														
Bifidobacteriaceae	39.6	(33.4)	40.9	38.6	-	58.8	28.1	0.03	46.7	32.6	-	48.0	19.3	0.03
*Bifidobacterium*	39.6	(33.4)	40.9	38.6	-	58.8	28.1	0.03	46.7	32.6	-	48.0	19.3	0.02
Coriobacteriaceae	1.4	(3.3)	0.3	2.3	0.07	2.2	0.9	-	2.0	0.8	-	1.7	0.6	-
*Eggerthella*	0.5	(1.0)	0.3	0.6	-	0.7	0.3	-	0.8	0.1	0.01	0.4	0.6	-
**Bacteroidetes**														
Bacteroidaceae	0.8	(1.5)	0.8	0.8	-	1.2	0.5	-	0.9	0.7	-	0.9	0.5	-
*Bacteroides*	0.8	(1.5)	0.8	0.8	-	1.2	0.5	-	0.9	0.7	-	0.9	0.5	-
**Firmicutes**														
Clostridiaceae	2.6	(7.2)	1.7	3.4	-	0.3	4.0	0.02	0.8	4.4	-	2.9	2.0	-
*Clostridium*	2.6	(7.2)	1.7	3.4	-	0.3	4.0	0.02	0.8	4.4	-	2.9	2.0	-
Enterococcaceae	1.5	(2.2)	1.7	1.3	-	0.9	1.8	-	0.7	2.3	0.09	1.6	1.1	-
*Enterococcus*	1.5	(2.2)	1.7	1.3	-	0.9	1.8	-	0.7	2.3	0.08	1.6	1.1	-
Erysipelotrichaceae	5.2	(8.7)	5.4	5.1	-	2.4	7.0	-	4.4	6.1	-	3.7	9.0	-
Lachnospiraceae	22.0	(22.0)	26.0	18.6	-	17.6	24.6	-	25.7	18.2	-	16.6	35.1	0.14
*Blautia*	3.0	(7.6)	5.4	0.9	-	0.6	4.4	-	0.7	5.2	-	0.9	8.1	-
*Coprococcus*	0.8	(2.6)	0.7	1.0	-	0.0	1.3	0.01	0.0	1.6	0.12	0.2	2.3	-
Peptostreptococcaceae	1.2	(2.0)	2.3	0.3	0.02	0.1	1.9	0.007	0.6	1.9	0.14	0.7	2.4	0.08
Ruminococcaceae	0.6	(1.1)	0.9	0.4	0.12	0.4	0.7	-	0.4	0.8	-	0.6	0.5	-
Streptococcaceae	5.7	(11.8)	2.5	8.4	-	3.3	7.1	-	3.0	8.4	-	4.9	7.6	-
*Streptococcus*	5.7	(11.8)	2.5	8.3	-	3.3	7.1	-	2.9	8.4	-	4.9	7.6	-
Veillonellaceae	4.7	(6.9)	5.2	4.3	-	1.2	6.8	0.02	1.6	7.8	0.02	3.4	7.7	-
*Veillonella*	4.6	(6.9)	5.0	4.3	-	1.2	6.7	0.02	1.6	7.6	0.03	3.4	7.7	-
**Verrucomicrobia**														
Verrucomicrobiaceae	0.6	(2.2)	0.9	0.3	0.06	0.4	0.7	-	0.3	0.9	-	0.8	0.0	0.01
*Akkermansia*	0.6	(2.2)	0.9	0.3	0.06	0.4	0.7	-	0.3	0.9	-	0.8	0.0	0.01
**Proteobacteria**														
Enterobacteriaceae	11.8	(11.1)	9.6	13.7	-	10.7	12.5	-	12.5	11.2	-	11.8	11.9	-
*Escherichia_Shigella*	10.5	(11.5)	8.8	11.9	-	10.4	10.5	-	12.3	8.7	-	10.4	10.7	-
Pasteurellaceae	0.2	(0.8)	0.1	0.3	-	0.0	0.4	0.09	0.1	0.4	-	0.2	0.2	-
*Haemophilus*	0.2	(0.8)	0.1	0.3	-	0.0	0.4	0.09	0.1	0.4	-	0.2	0.2	-

SD, standard deviation. Differential abundance test conducted using Metastats algorithm. P-values >0.15 not shown. Taxa were excluded from this analysis if they did not exceed 1% relative abundance in at least 1 sample, or were not present in at least 3 infants.

Because of its reported association with atopic outcomes [16], we also performed targeted qPCR analyses to detect colonization by *C. difficile* (a member of the family Peptostreptococcaceae). Consistent with the antithetical associations observed for Peptostreptococcaceae relative abundance, *C. difficile* prevalence tended to be higher among infants with pets, and lower among infants with siblings: *C. difficile* was detected in 67% of infants with pets, compared with 22% of infants without pets (p = 0.09), and 31% of infants with older siblings, compared with 73% of first-born infants (p = 0.10). Comparison of qPCR and sequencing results revealed that sequencing-based analyses were highly specific and moderately sensitive for *C. difficile*. Two of seven Peptostreptococcaceae OTUs could be further classified as different strains of *C. difficile*; these OTUs were absent in all infants testing negative by qPCR (100% specificity), and were detected in 8 of 12 infants testing positive (67% sensitivity).

### Biodiversity

**Figure 2:**
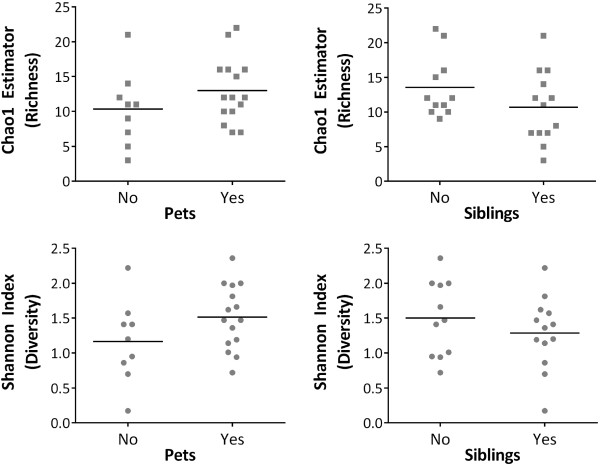
**Richness and diversity of infant fecal microbiota according to the presence of household pets and siblings.** Richness (Chao1 estimator) and diversity (Shannon Index) were calculated from 16S rRNA sequencing data (see Methods and Table 3).

Overall, the mean rarefied Chao1 score for species richness (which evaluates the number of different species present) was 12, ranging from 3 to 22. The average Shannon diversity index (which accounts for both species richness and evenness) was 1.38, ranging from 0.17 to 2.36. Opposite trends in biodiversity were observed with respect to pets versus siblings (Figure 2 and Table 3). Although differences did not reach statistical significance, infants with pets tended to have increased microbiota richness and diversity, whereas these measures tended to be decreased in infants with older siblings.

**Table 3:** Infant fecal microbiota richness and diversity according to household pets and siblings

	**N**	**Chao1 Estimate:**			**Shannon index:**		
		**Richness**			**Diversity**		
		**Mean**	**(SD)**	**p**	**Mean**	**(SD)**	**p**
**Overall**	24	12	(4.5)		1.38	(0.5)	
**Pets**							
No	9	10.3	(5.3)	0.21	1.16	(0.59)	0.12
Yes	15	13.0	(4.6)		1.51	(0.46)	
**Older siblings**							
No	11	13.5	(4.5)	0.16	1.50	(0.54)	0.34
Yes	13	10.7	(5.1)		1.29	(0.52)	

SD, standard deviation. Comparison by unpaired, 2-sided t-test.

## Discussion

**Figure 3:**
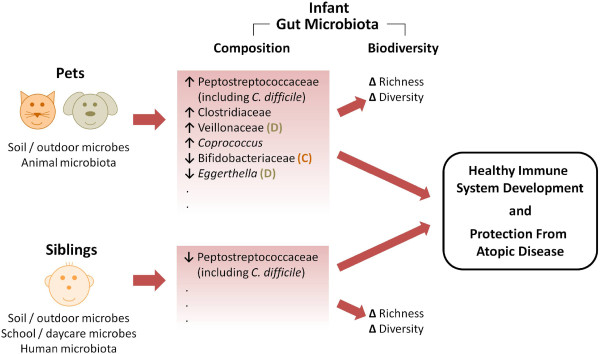
**Model for the possible influence of pets and siblings on infant gut microbiota and subsequent development of atopic disease.** Household pets (D, dogs; C, cats) and siblings increase infant exposure to environmental microbes, promoting enrichment for distinct combinations of organisms within the gut microbiota; overall richness and diversity are also impacted. Despite favoring different microbiota profiles, the net effect of both pets and siblings is to promote healthy immune system development and protect against atopic disease. Further research is required to characterize the underlying biological mechanisms.

Using a comprehensive and culture-independent approach, we have characterized the impact of household pets and siblings on the composition and diversity of infant gut microbiota. Our findings suggest that these two traditionally protective “hygiene hypothesis” factors exert distinct effects on microbiota diversity, and select for different assemblages of gut microbes (Figure 3). These results imply that the “microflora hypothesis” of allergic disease is likely a function of complex changes in microbiota composition, rather than simplified variations in overall diversity.

Although no clear consensus has been reached, many studies have reported inverse associations between early-life pet exposure and allergic disease. In a questionnaire study of over 2000 Swedish children, exposure to pets during the first year of life was associated with a lower prevalence of allergic rhinitis and asthma at school age [2]. Similarly in an American prospective birth cohort, exposure to 2 or more dogs or cats in the first year of life was found to reduce subsequent risk of allergic sensitization to multiple allergens during childhood [1]. Whether these associations can be attributed to changes in the infant gut microbiota remains to be seen. Sharing of specific microbes between pet and owner has been occasionally reported in adults [30-32], but colonization of the infant gut has rarely been studied in relation to pets. Using targeted methods to identify specific organisms, it has been reported that the presence of household pets does not influence colonization by *Lactobacillus*, *E. coli*, or *C. difficile*[23,33,34]. Ours is the first comprehensive profiling study to address the impact of pets on the infant gut microbiome as a whole.

Similar to household pets, the presence of older siblings has been associated with a reduced risk of atopic disease. Indeed, it was David Strachan’s landmark paper on the inverse association between hay fever and household size (especially older siblings) that inspired the hygiene hypothesis [5]. Similar associations have since been reported around the world for both asthma [2] and food allergy [21]. Although frequently suggested as the underlying mechanism for these associations, there is only limited evidence that contact with siblings alters the infant gut microbiota. Using targeted or culture-based methods, others have shown that infants with older siblings have slightly higher numbers of Bifidobacteria [23], delayed colonization by *Clostridium* species [22], and increased colonization by *Staphylococcus aureus*[35], with no difference in *C. difficile* colonization [28]. Until now, no study has reported the impact of siblings on the overall community structure of the gut microbiota.

Thus, despite the vast amount of evidence that household pets and siblings protect against allergic disease, and the widespread consensus that microbial exposure underlies these associations, ours is the first study to directly assess the impact of pets and siblings on the community composition and diversity of infant gut microbiota. We observed that while microbiota richness and diversity tended to be increased in infants living with pets, the opposite was true for infants with older siblings. Pets and siblings also appeared to select for distinct assemblages of gut microbes, possibly because they harbor and transmit different organisms (e.g. microbes from the outdoor environment, schools or daycares, and organisms from the normal animal or human microbiota). Transmission of microbes to the infant could occur through direct contact, or through intermediate reservoirs in the home environment (such as house dust or the maternal microbiome) [36,37]. Also, in contrast to a previous study [28], we found that *C. difficile* tended to be more prevalent among infants living with pets, and less prevalent among infants with siblings. Despite these apparently opposing influences, both pets and siblings are presumably protective against allergic disease (although this remains to be confirmed in our cohort, once the participants are old enough to evaluate atopic outcomes).

Collectively, this evidence suggests that simplified measures of microbiota diversity may be insufficient or even misleading, for the purposes of evaluating atopic disease risk and related biological mechanisms. Indeed, conflicting reports have indicated that both low and high infant gut microbiota diversity are associated with atopic eczema [7,38], illustrating the limited clinical value of these measures. The observation that two protective exposures apparently select for different combinations of microbes indicates that multiple “healthy” microbiota profiles are possible, perhaps due to functional redundancy among different organisms. As others have suggested, this functional redundancy may obviate the need for “keystone species” and high microbial diversity [15,39].

As the microbiota of domestic dogs, cats and other companion animals continue to become better defined by modern methods [40-42], the ability to resolve their microbial contributions to other microbial communities will improve. Because of the great degree of overlap at the genus-level between human- and pet-associated microbiota, sequencing approaches that yield species-level or even strain-level resolution are ultimately needed to differentiate these communities. In the meantime, algorithms to establish robust associations have already been demonstrated using sequence data of coarser taxonomic resolution [43-45]).

The major strengths of this study are the application of modern high-throughput sequencing technology to profile the gut microbiota of healthy infants, and the prospective collection of detailed exposure data. Our combined use of 16S rRNA sequencing and targeted qPCR analysis permitted the integration of reliable species-level data into the framework of comprehensive, culture-independent microbiome analysis. In addition, our finding that cats and dogs seem to favour enrichment of different organisms in the gut microbiota indicates that “pet exposure” is complex and should be defined as specifically as possible in population health studies in order to support future analyses.

The main limitation of this study is sample size; despite being relatively large among infant gut microbiota studies (many of which have comprised fewer than 15 infants [46-52]), we had insufficient power to detect differences according to type of pet or number of siblings. Also, we could not adjust for the simultaneous effects of multiple exposures; since the majority (>80%) of infants without siblings in this cohort happened to live with pets, it is possible that pet exposure may be driving the sibling-associated differences observed in the current study. Further research is required to establish the independent and combined effects of these and other exposures, including potential confounders such as antibiotic use, caesarean delivery or breastfeeding [53,54]. These deficiencies will be addressed in our ongoing research within the full CHILD cohort, where longitudinal sampling and clinical evaluation of over 2000 infants is anticipated, and multivariate analyses will be possible.

## Conclusions

In summary, this study provides new evidence that early-life exposure to pets and siblings may influence the overall diversity and composition of the infant gut microbiota, with potential implications for the development of atopy (Figure 3). Allergic diseases have reached epidemic proportions in recent decades, and our research supports the hypothesis that modifiable environmental exposures may be responsible, through their impact on the gut microbiota. Our findings also emphasize the complexity of these associations, and underscore the need for further research.

## Abbreviations

CHILD: Canadian Healthy Infant Longitudinal Development Study; OTU: Operational taxonomic unit; qPCR: Quantitative polymerase chain reaction.

## Competing interests

The authors declare that they have no competing interests.

## Authors’ contributions

MBA analyzed data and drafted manuscript; TK processed samples; HM processed sequence data; CJF provided data interpretation; ABB coordinated subject recruitment and sample/data collection; DSG, JAS & ALK conceptualized and designed the study; MRS and the CHILD investigators contributed to the design/execution of the CHILD birth cohort. All authors contributed to data interpretation, and critically reviewed and approved the manuscript.

## Supplementary Material

Additional file 1 Prevalence of dominant bacterial taxa in infant stool, according to household pets and siblings. Click here for file
