# Supplementary material for: Infant gut microbiota and the hygiene hypothesis of allergic disease: impact of household pets and siblings on microbiota composition and diversity
Source: Allergy Asthma Clin Immunol. 2013 Apr 22;9(1):15. doi: 10.1186/1710-1492-9-15 (PMC3655107; doi:10.1186/1710-1492-9-15)
Supplement: Additional file 1 — Prevalence of dominant bacterial taxa in infant stool, according to household pets and siblings. [file 1710-1492-9-15-S1.doc]

**Table S1. Prevalence of dominant bacterial taxa in infant stool, according to household pets and siblings.**

|  |  | **Overall** | |  | **Older Siblings** | | |  | **Pets** | | |  | **Dog** | | |  | **Cat** | | |
| --- | --- | --- | --- | --- | --- | --- | --- | --- | --- | --- | --- | --- | --- | --- | --- | --- | --- | --- | --- |
| Variable |  | N = 24 | |  | **No** | **Yes** | **p** |  | **No** | **Yes** | **p** |  | **No** | **Yes** | **p** |  | **No** | **Yes** | **p** |
|  |  | (n) | % |  | N=11 | N=13 |  |  | N=9 | N=15 |  |  | N=12 | N=12 |  |  | N=17 | N=7 |  |
| **Actinobacteria** |  |  |  |  |  |  |  |  |  |  |  |  |  |  |  |  |  |  |  |
| Bifidobacteriaceae |  | (18) | 75.0 |  | 81.8 | 69.2 | - |  | 88.9 | 66.7 | - |  | 83.3 | 66.7 | - |  | 76.5 | 71.4 | - |
| *Bifidobacterium* |  | (18) | 75.0 |  | 81.8 | 69.2 | - |  | 88.9 | 66.7 | - |  | 83.3 | 66.7 | - |  | 76.5 | 71.4 | - |
| Coriobacteriaceae |  | (11) | 45.8 |  | 36.4 | 53.8 | - |  | 66.7 | 33.3 | - |  | 58.3 | 33.3 | - |  | 52.9 | 28.6 | - |
| *Eggerthella* |  | (8) | 33.3 |  | 27.3 | 38.5 | - |  | 44.4 | 26.7 | - |  | 41.7 | 25.0 | - |  | 35.3 | 28.6 | - |
|  |  |  |  |  |  |  |  |  |  |  |  |  |  |  |  |  |  |  |  |
| **Bacteroidetes** |  |  |  |  |  |  |  |  |  |  |  |  |  |  |  |  |  |  |  |
| Bacteroidaceae |  | (9) | 37.5 |  | 54.5 | 23.1 | - |  | 44.4 | 33.3 | - |  | 33.3 | 41.7 | - |  | 47.1 | 14.3 | - |
| *Bacteroides* |  | (9) | 37.5 |  | 54.5 | 23.1 | - |  | 44.4 | 33.3 | - |  | 33.3 | 41.7 | - |  | 47.1 | 14.3 | - |
|  |  |  |  |  |  |  |  |  |  |  |  |  |  |  |  |  |  |  |  |
| **Firmicutes** |  |  |  |  |  |  |  |  |  |  |  |  |  |  |  |  |  |  |  |
| Clostridiaceae |  | (12) | 50.0 |  | 72.7 | 30.8 | 0.10 |  | 33.3 | 60.0 | - |  | 41.7 | 58.3 | - |  | 41.2 | 71.4 | - |
| *Clostridium* |  | (12) | 50.0 |  | 72.7 | 30.8 | 0.10 |  | 33.3 | 60.0 | - |  | 41.7 | 58.3 | - |  | 41.2 | 71.4 | - |
| Enterococcaceae |  | (15) | 62.5 |  | 63.6 | 61.5 | - |  | 55.6 | 66.7 | - |  | 50.0 | 75.0 | - |  | 70.6 | 42.9 | - |
| *Enterococcus* |  | (15) | 62.5 |  | 63.6 | 61.5 | - |  | 55.6 | 66.7 | - |  | 50.0 | 75.0 | - |  | 70.6 | 42.9 | - |
| Erysipelotrichaceae |  | (14) | 58.3 |  | 45.5 | 69.2 | - |  | 55.6 | 60.0 | - |  | 66.7 | 50.0 | - |  | 52.9 | 71.4 | - |
| Lachnospiraceae |  | (22) | 91.7 |  | 100.0 | 84.6 | - |  | 77.8 | 100.0 | 0.13 |  | 83.3 | 100.0 | - |  | 88.2 | 100.0 | - |
| *Blautia* |  | (6) | 25.0 |  | 36.4 | 15.4 | - |  | 22.2 | 26.7 | - |  | 25.0 | 25.0 | - |  | 17.6 | 42.9 | - |
| *Coprococcus* |  | (4) | 16.7 |  | 18.2 | 15.4 | - |  | 0.0 | 26.7 | - |  | 8.3 | 25.0 | - |  | 5.9 | 42.9 | 0.06 |
| Peptostreptococcaceae |  | (11) | 45.8 |  | 63.6 | 30.8 | - |  | 11.1 | 66.7 | 0.01 |  | 25.0 | 66.7 | 0.10 |  | 35.3 | 71.4 | - |
| Ruminococcaceae |  | (8) | 33.3 |  | 45.5 | 23.1 | - |  | 22.2 | 40.0 | - |  | 25.0 | 41.7 | - |  | 29.4 | 42.9 | - |
| Streptococcaceae |  | (20) | 83.3 |  | 81.8 | 84.6 | - |  | 88.9 | 80.0 | - |  | 91.7 | 75.0 | - |  | 76.5 | 100.0 | - |
| *Streptococcus* |  | (20) | 83.3 |  | 81.8 | 84.6 | - |  | 88.9 | 80.0 | - |  | 91.7 | 75.0 | - |  | 76.5 | 100.0 | - |
| Veillonellaceae |  | (17) | 70.8 |  | 90.9 | 53.8 | 0.08 |  | 55.6 | 80.0 | - |  | 58.3 | 83.3 | - |  | 70.6 | 71.4 | - |
| *Veillonella* |  | (16) | 66.7 |  | 81.8 | 53.8 | - |  | 55.6 | 73.3 | - |  | 58.3 | 75.0 | - |  | 64.7 | 71.4 | - |
|  |  |  |  |  |  |  |  |  |  |  |  |  |  |  |  |  |  |  |  |
| **Verrucomicrobia** |  |  |  |  |  |  |  |  |  |  |  |  |  |  |  |  |  |  |  |
| Verrucomicrobiaceae |  | (3) | 12.5 |  | 9.1 | 15.4 | - |  | 11.1 | 13.3 | - |  | 16.7 | 8.3 | - |  | 11.8 | 14.3 | - |
| *Akkermansia* |  | (3) | 12.5 |  | 9.1 | 15.4 | - |  | 11.1 | 13.3 | - |  | 16.7 | 8.3 | - |  | 11.8 | 14.3 | - |
|  |  |  |  |  |  |  |  |  |  |  |  |  |  |  |  |  |  |  |  |
| **Proteobacteria** |  |  |  |  |  |  |  |  |  |  |  |  |  |  |  |  |  |  |  |
| Enterobacteriaceae |  | (24) | 100.0 |  | 100.0 | 100.0 | - |  | 100.0 | 100.0 | - |  | 100.0 | 100.0 | - |  | 100.0 | 100.0 | - |
| *Escherichia_Shigella* |  | (20) | 83.3 |  | 81.8 | 84.6 | - |  | 88.9 | 80.0 | - |  | 91.7 | 75.0 | - |  | 88.2 | 71.4 | - |
| Pasteurellaceae |  | (3) | 12.5 |  | 9.1 | 15.4 | - |  | 0.0 | 20.0 | - |  | 8.3 | 16.7 | - |  | 11.8 | 14.3 | - |
| *Haemophilus* |  | (3) | 12.5 |  | 9.1 | 15.4 | - |  | 0.0 | 20.0 | - |  | 8.3 | 16.7 | - |  | 11.8 | 14.3 | - |
|  |  |  |  |  |  |  |  |  |  |  |  |  |  |  |  |  |  |  |  |
| Comparisons by Fisher exact test. P-values >0.15 not shown. Taxa were excluded from this analysis if they did not exceed 1% relative abundance in at least 1 sample, or were not present in at least 3 infants. | | | | | | | | | | | | | | | | | | | |
